# Hospital volume and the risk of revision in Oxford unicompartmental knee arthroplasty in the Nordic countries -an observational study of 14,496 cases

**DOI:** 10.1186/s12891-017-1750-7

**Published:** 2017-09-07

**Authors:** Mona Badawy, Anne M. Fenstad, Christoffer A. Bartz-Johannessen, Kari Indrekvam, Leif I. Havelin, Otto Robertsson, Annette W-Dahl, Antti Eskelinen, Keijo Mäkelä, Alma B. Pedersen, Henrik M. Schrøder, Ove Furnes

**Affiliations:** 1 Coastal Hospital, 5253 Hagavik, Norway; 2 The Norwegian Arthroplasty Register, Department of Orthopaedic Surgery, Haukeland University Hospital, Bergen, Norway; 3 Department of Clinical Medicine, Institute of Medicine and Dentistry, University of Bergen, Bergen, Norway; 4 The Swedish Knee Arthroplasty Register, Lund, Sweden; 5 Department of Clinical Sciences, Lund University Faculty of Medicine, Orthopedics, Lund, Sweden; 6 The Coxa Hospital for Joint Replacement, Tampere, Finland; 7 Department of Orthopaedics and Traumatology, Turku University Hospital, Turku, Finland; 8 The Danish Knee Arthroplasty Register, Aarhus, Denmark; 9 Department of Clinical Epidemiology, Aarhus University Hospital, Aarhus, Denmark; 10 Department of Orthopaedic surgery, Næstved Hospital, Næstved, Denmark

**Keywords:** Knee, Osteoarthritis, Arthroplasty, Unicompartmental, Procedure volume, Revision causes

## Abstract

**Background:**

High procedure volume and dedication to unicompartmental knee arthroplasty (UKA) has been suggested to improve revision rates. This study aimed to quantify the annual hospital volume effect on revision risk in Oxfordu﻿ ﻿nicompartmental knee arthroplasty in the Nordic countries.

**Methods:**

14,496 cases of cemented medial Oxford III UKA were identified in 126 hospitals in the four countries included in the Nordic Arthroplasty Register Association (NARA) database from 2000 to 2012. Hospitals were divided by quartiles into 4 annual procedure volume groups (≤11, 12-23, 24-43 and ≥44). The outcome was revision risk after 2 and 10 years calculated using Kaplan Meier method. Multivariate Cox regression analysis was used to assess the Hazard Ratio (HR) of any revision due to specific reasons with 95% confidence intervals (CI).

**Results:**

The implant survival was 80% at 10 years in the volume group ≤11 procedures per year compared to 83% in other volume groups. The HR adjusted for age category, sex, year of surgery and nation was 0.87 (95% CI: 0.76-0.99, *p* = 0.036) for the group 12-23 procedures per year, 0.78 (95% CI: 0.68-0.91, *p* = 0.002) for the group 24-43 procedures per year and 0.82 (95% CI: 0.70-0.94, *p* = 0.006) for the group ≥44 procedures per year compared to the low volume group. Log-rank test was *p* = 0.003. The risk of revision for unexplained pain was 40-50% higher in the low compared with other volume groups.

**Conclusion:**

Low volume hospitals performing ≤11 Oxford III UKAs per year were associated with an increased risk of revision compared to higher volume hospitals, and unexplained pain as revision cause was more common in low volume hospitals.

## Background

The Oxford unicompartmental knee arthroplasty (UKA) has been investigated in numerous studies due to the deviant results comparing registry results to studies from high volume centers and surgeons. Data from national registries show a significantly higher revision rate for both short and long term results for UKA than for total knee arthroplasty (TKA) [1–5]. Other studies from high-volume Oxford developing centers, however, show excellent long-term results [6, 7]. The existing variability in practice regarding indication and usage of UKA results in low volumes in hospitals using strict criteria [8], and higher volumes in hospitals offering UKA to patients using less strict criteria [9]. The Nordic Arthroplasty Register Association is a collaboration of arthroplasty registers in Sweden, Denmark, Norway and Finland established in 2007. The cooperation has produced a common defined set of variables agreed upon, enabling analyses of larger statistical material [10]. This is an advantage especially for uncommon methods and procedures, such as the UKA constituting only 11% of the knee arthroplasties in the Nordic countries [11]. The advantage of a registry study for our purpose was the representation of all surgeons in all hospitals in Sweden, Denmark, Finland and Norway resulting in more generalizable findings. The UKA is utilized at similar lower percentage than TKA in the majority of countries with registries worldwide for the treatment of osteoarthritis [2, 12]. The aim of this study was to investigate how the patient risk for revision surgery after Oxford III UKA varied as a function of hospital procedure volume. Adding to the analyses for all causes of revision, the second objective was to assess any differences in the proportion of the specific causes of revision according to volume groups.

## Methods

### Data sources

We used the NARA database, containing a common defined code set to identify patients undergoing primary cemented medial Oxford III UKA between January 1, 2000 and December 31, 2012 in this population-based register study [11, 13]. Every year all uniform variables from each national register are re-coded according to common definitions and anonymized and then merged into the NARA database. The linkage between primary procedure and subsequent revision or death on individual data is performed in each national register before merged into the NARA database. The first studies focused on differences in patient demographics, surgical methods and implant brands [10, 11, 14]. The main purpose of NARA was the ability to analyze a larger statistical material, which is an advantage especially for uncommon methods and implants. It reflects the current practice in 4 different countries. The knee dataset currently includes 390,525 primary knee arthroplasty operations performed during 1995-2012 [13]. The Oxford UKA was the most commonly registered UKA implant in the NARA.

### Study population

**Fig. 1:**
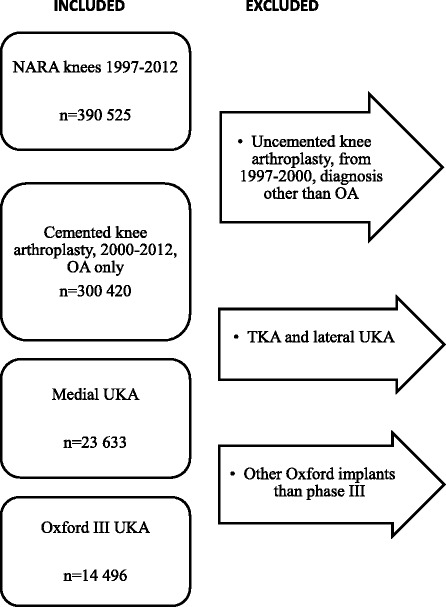
14,496 cemented medial Oxford III UKA from 2000 to 2012 were selected for inclusion in this study. Other diagnoses than osteoarthritis were excluded. Abbreviations: NARA = Nordic Arthroplasty Register Association, OA = osteoarthritis

Implant brand and type could be a source of confounding in comparison to revision rate according to hospital, and therefore all other brands and types than Oxford III UKA were excluded. Diagnoses other than osteoarthritis (OA) were excluded as inflammatory disease is a contraindication in UKA. The inclusion criteria for this study, to obtain comparable groups for analysis, are shown in the flowchart (Fig. 1). In NARA revision is defined as removal/exchange/addition of one or more implant component(s) and is linked to the primary procedure by the unique national identification number of the patient.

We identified 4211 (29.0%) Oxford III implants in Denmark in 32 different hospitals: 2218 (15.3%) in Sweden distributed among 18 hospitals, 3910 (27.0%) in Finland in 41 hospitals and 4157 (28.7%) in Norway in 35 hospitals (Table 1). The inclusion of bilateral knee arthroplasty can be a violation of the assumption of independent observations in survival analyses, but studies have shown that the effect is minor regarding statistical precision for survival analysis of knee replacements [15]. In this study, 14% of the patients had bilateral knee arthroplasty.

**Table 1:** Patient and procedure characteristics of 14,496 cemented medial Oxford III unicompartmental knee arthroplasty according to four hospital volume categories with the diagnosis osteoarthritis from 2000 to 2012

	Annual hospital volume groups				
	≤11	12-23	24-43	≥44	
					*p*-values
No of procedures	3528	3759	3533	3676	
Men %	42	43	44	44	0.17
Age^median^ (range)	62 (28-94)	63 (34-93)	65 (33-94)	65 (33-95)	
Age group n (%)					
<55	731 (21)	652 (17)	501 (14)	540 (15)	<0.001
55-64	1471 (42)	1469 (39)	1199 (34)	1339 (36)	
65-74	946 (27)	1169 (31)	1251 (35)	1240 (34)	
≥75	380 (11)	469 (13)	582 (17)	566 (15)	
Year of surgery					
2000-03	962 (27)	826 (22)	399 (11)	475 (13)	<0.001
2004-06	928 (26)	1061 (28)	562 (16)	1113 (30)	
2007-09	925 (26)	1281 (32)	1349 (38)	900 (25)	
2010-12	713 (20)	654 (18)	1223 (35)	1188 (32)	
Nation n (%)					
Denmark (4211)	558 (16)	615 (16)	1118 (32)	1920 (52)	<0.001
Norway (4157)	1273 (36)	1551 (41)	1147 (33)	186 (5)	
Sweden (2218)	460 (13)	561 (15)	508 (14)	689 (19)	
Finland (3920)	1237 (35)	1032 (28)	760 (21)	881 (24)	

### Exposure

All Oxford III UKA procedures were entered into one of four different annual hospital volume groups. We used quartiles to divide into equal numbered volume groups; ≤11, 12-23, 24-43 and ≥44 procedures per year. Hospitals with inconsistent procedure volume over time may have contributed to different volume groups according to the number of procedures at their hospital in the year of surgery. Thus, for each hospital each year was examined individually. This categorization of the exposure assumes that unspecified hospital-level effects are trumped by a potential volume effect on revision rates. Revision due to any reason as well as specific causes for revision was analysed.

### Statistics

Survival analyses were performed with any revision of the implant as endpoint. Kaplan Meier cumulative survival at 2 and 10 years was reported. A 2 year follow-up was chosen to assess early revisions. The follow-up started at the day of primary UKA procedure and ended at the day of first revision, death, emigration or the end of follow-up time (December 31st 2012). The two highest volume groups had shorter follow-up compared to the lower (chi-square test *p*-value <0.001). Log-rank test was performed, *p* = 0.003. Differences for categorical variables such as sex, age categories, year of surgery and nations were assessed by Pearson’s chi-squared test. Any *p*-values less than 0.05 were considered significant. To estimate differences in continuous variables the student t-test was used.

The Cox regression model was used to calculate Hazard Ratios (HR) with 95% confidence interval (CI) for the 10 year follow-up period to investigate the association between four hospital procedure volume groups and implant survival time. *P*-values were presented relative to the lowest volume group (≤ 11). All p-values less than 0.05 were considered to be statistically significant. The Cox model included sex, age category, year of surgery, nation and hospital volume. Death is to be considered a possible competing risk to revision. We studied the influence of death by performing a competing risk analysis using the statistical software R [16, 17]. The results for the volume groups did not change significantly when accounting for death as a competing risk for revision (Table 2). Cox regression analyses were made for the different confounding variables and are presented in Table 3.

**Table 2:** Results from survival and Cox regression analyses on hospital volume for 14,496 cemented medial Oxford III unicompartmental knee arthroplasty in NARA 2000-2012

Annual hospital volume groups	Number of procedures	Number of revisions (%)	Number of deaths^a^ (%)	K-M 2-year survival (95%CI)	K-M 10-year survival (95%CI)	Cox Regression Unadjusted HR(95%CI) 10 years	p-value	Cox Regression Adjusted RR(95%CI) 10 years	p-value
≤11	3528	481 (13.6)	231 (6.5)	93 (92.4-94.0)	80 (78.0-82.0)	1.0 (ref)		1.0 (ref)	
12-23	3759	429 (11.4)	237 (6.3)	95 (94.1-95.7)	83 (80.8-84.4)	0.85 (0.75-0.97)	0.017	0.87 (0.76-0.99)	0.036
24-43	3533	293 (8.3)	185 (5.2)	95 (94.1-95.7)	83 (80.2-86.2)	0.78 (0.67-0.90)	0.001	0.78 (0.68-0.91)	0.002
≥44	3676	351 (9.5)	227 (6.2)	95 (94.3-95.9)	83 (80.7-85.5)	0.82 (0.72-0.95)	0.006	0.82 (0.70-0.94)	0.006

*K-M* Kaplan-Meier estimated cumulative survival at 2 and 10 years (%)

*HR* Hazard Ratio; with adjustment for age category, sex, year of surgery and nation

*CI* confidence interval

*Ref* reference

^a^No statistical significant differences in proportion of deaths within the groups, p-value equal to 0.11

**Table 3:** Cox proportional survival model with Hazard Ratios (HR) adjusted for age, sex, year of surgery and nation as covariates with 95% CI (confidence interval) for all reasons for revision up to 10 years after primary surgery

Variables	No of procedures	HR(95%CI)	*p-*value
Age group			
55-64	5469	1.0 (ref)	
< 55	2424	1.3 (1.1-1.5)	<0.001
65-74	4606	0.8 (0.7-0.9)	<0.001
≥ 75	1997	0.7 (0.6-0.8)	<0.001
Sex			
Male	6272	1.0 (ref)	
Female	8224	1.0 (0.9-1.1)	0.6
Year of surgery			
2000-03	2662	1.0 (ref)	
2004-06	3664	1.2 (1.0-1.3)	0.04
2007-09	4392	1.2 (1.0-1.4)	0.02
2010-12	3778	1.3 (1.1-1.6)	0.004
Nation			
Sweden	2218	1.0 (ref)	
Denmark	4211	1.4 (1.2-1.7)	<0.001
Norway	4157	1.2 (1.0-1.5)	0.01
Finland	3910	1.2 (1.0-1.4)	0.05

The various reasons for revision were organized hierarchically with infection first and unexplained pain last, as shown in Table 4. Loosening and wear were second in the list and instability and dislocation third. The group ‘other reasons’ contained new diseases occurring in the joint such as osteoarthritis or osteonecrosis laterally or joint fibrosis with stiffness. Surgical errors such as incorrect sizing of components were also included in this group. When more than one reason was reported, the top reason in the hierarchy was used as endpoint in the analyses. Pain as a cause of revision was used as endpoint only when pain was the only reason reported. HR with 95% CI was reported for different revision causes with 10 years follow-up. The proportional hazards assumption of the Cox model was tested based on log-minus-log plot and found to be valid. SPSS version 23 and R statistical software package version 3.2.1 were used for the statistical analyses.

**Table 4:** Revisions causes with 10 years follow up. Hazard Ratios with confidence intervals for different hospital volumes, adjusted for sex, age category, year of surgery and nation

Annual hospital volume group	Number of procedures	Number of revisions	Revision causes with adjusted Hazard Ratios (95% confidence interval)					
			Infection	Loosening/Wear	Instability/Dislocation	Unexplained pain	Other reasons	Unknown reasons
		1519	*n* = 57	*n* = 545	*n* = 104	*n* = 273	*n* = 498	*n* = 42
≤11	3528	465	1.0 (ref)	1.0 (ref)	1.0 (ref)	1.0 (ref)	1.0 (ref)	1.0 (ref)
12-23	3759	420	1.02 (0.49-2.16)	1.04 (0.83-1.29)	1.66 (1.00-2.76)	0.54 (0.39-0.75)	0.72 (0.57-0.91)	1.90 (0.83-4.39)
24-43	3533	292	0.92 (0.41-2.02)	0.80 (0.61-1.04)	1.12 (0.61-2.05)	0.64 (0.46-0.89)	0.80 (0.62-1.04)	1.12 (0.44-2.86)
≥44	3676	342	1.20 (0.54-2.64)	0.93 (0.73-1.19)	1.15 (0.58-2.28)	0.56 (0.39-0.80)	0.77 (0.60-0.99)	0.47 (0.14-1.51)
Number of revisions in each volume group:								
≤11			13	157	23	96	168	8
12-23			15	166	43	60	118	18
24-43			13	93	21	61	93	11
≥44			16	129	17	56	119	5

*n* numbers

*ref* reference

## Results

126 hospitals performed 14,496 cemented medial Oxford III UKA from 2000 to 2012 in the 4 Nordic countries. Demographics and patient characteristics are shown in Table 1. The median number of procedures performed annually by a hospital was 23 (IQR (inter quartile range) =12-44). The median annual procedure volume per hospital in Denmark was 41 (IQR = 23-61), 27 (IQR = 13-48) in Sweden, 18 (IQR = 9-36) in Finland and 17 (IQR = 10-26) in Norway. The most common annual hospital volume was 1 per year, the second and third most common annual procedure volume was 2 and 3 per year respectively.

The Kaplan Meier 2 year survival was 95% for the three hospitals groups with annual procedure volume > 11 and 93% for the hospitals performing ≤11 Oxford III UKA per year. The Kaplan Meier estimated survival had dropped to 80% at 10 years follow up with poorest result for the ≤11 per year group (Table 2). The three hospital volume groups of >11 had an estimated survival of 83% at 10 years. The Log-rank test was statistically significant with *p* = 0.003.

**Fig. 2:**
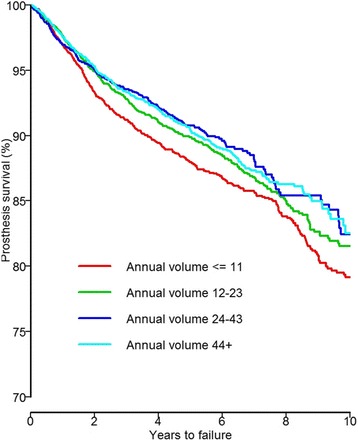
Cox regression survival curve adjusted for age, sex, year of surgery and nation

In the Cox regression model, the high volume groups (≥44 procedures per year) had a lower risk of any revision during the entire follow-up time of 10 years compared with the lowest volume group (≤ 11 procedures per year) according to adjusted HR = 0.82 (95% CI 0.70-0.94, *p* = 0.006). Similarly, the adjusted HRs were 0.78 (95% CI 0.68-0.91, *p* = 0.002) for the group performing 24-43 procedures per year and 0.87 (95% CI 0.76-0.99, *p* = 0.036) for the group performing 12-23 procedures per year compared to the lowest volume group (Table 2, Fig. 2).

In the multivariable survival model we found inferior results for the youngest age group <55 with Hazard Ratio HR = 1.29 (95% CI 1.13-1.47, p = <0.001) with 55-64 as reference. The ≥75 age group showed better results; HR = 0.65 (95% CI 0.55-0.78, *p* < 0.001) (Table 3, Table 5). Gender was not found to influence the results. There seems to be a deterioration in results in the more recent years of surgery (HR = 1.33 (95%CI 1.10-1.62)). Denmark had statistically significant higher relative risk (HR = 1.41 (95%CI 1.19-1.68, p < 0.001)) compared to Sweden as reference. Similarly, Norway had HR = 1.24 (95%CI 1.05-1.47, *p* = 0.01). Finland had HR = 1.18 (95%CI 1.00-1.39, *p* = 0.05) (Table 3).

**Table 5:** Results from Kaplan Meier 10 year survival analysis for age as stratification variable according to volume groups with 95% confidence interval

	Age groups				
Annual Hospital Volume	< 55 yrs. *n* = 2424	55-64 *n* = 5469	65-74 *n* = 4606	≥75 *n* = 1997	For all ages (table 2) *n* = 14,496
≤11	74 (69.4-78.2)	79 (76.4-82.4)	82 (78.7-85.9)	89 (84.2-94.2)	80 (78.0-82.0)
12-23	74 (67.3-79.7)	81 (78.1-84.1)	87 (84.4-90.0)	87 (82.0-91.2)	83 (80.8-84.4)
24-43	77 (68.8-84.6)	83 (77.5-87.5)	84 (78.7-88.7)	90 (85.5-93.5)	83 (80.2-86.2)
≥44	79 (69.8-88.2)	81 (77.6-85.2)	84 (79.3-88.5)	90 (86.5-92.9)	83 (80.7-85.5)

*n* numbers

### Revision causes

The distribution of revision causes among the 1519 revised cemented medial Oxford III implants from 2000 to 2012—according to hospital volume—is shown in Table 4. We found a difference in the risk of revision for unexplained pain among the volume groups. The volume groups performing >11 Oxford III UKA per year revised 40-50% fewer patients for unexplained pain than the lowest volume hospitals (≤11 per year). The other revision causes did not show any statistically significant differences between the groups (Table 4).

## Discussion

In this large population based study based on 14,496 cemented medial Oxford III unicompartmental knee arthroplasty performed in four Scandinavian countries; we showed that high procedure volumes (>11 procedures per year) were associated with a decreased risk for revision.

This study contributes to the knowledge of other previously published results. There are available studies on the impact of procedure volume in UKA, and the common denominator is the Oxford implant since its usage is widespread. The Swedish study from 2001 found that performing less than 23 UKA per year was associated with a higher risk of revision [18], whereas Baker et al. [19] suggested a minimum annual volume of 13. Our previous study from Norway indicated fewer revisions with an annual caseload of more than 40 [20]. A study from the National Joint Registry of England and Wales (NJR) regarding determinants of revision following UKA supported the importance of experience measured at the unit level as well, and also favoring consultants rather than trainees [21]. A recent study from the NJR recommended surgeons to perform at least 20% of their knee arthroplasties as UKAs to achieve lower rates of revisions [22]. They also found that 81.4% of the surgeons performed less than 10 UKA per year. This corresponds to our findings of extreme skewness with dominance of low-volume performance. Some registers on the other hand recommend the use of fewer UKA due to higher failure rates [23]. Our study from 4 countries suggests a minimum hospital volume per hospital of 11. However, considering the variety of the previously mentioned studies and results, a threshold value of 11 per year could be considered a conservative value.

Our study included data from 4 different national registers with multiple surgeons and hospitals with varying experience and volume, suggesting high external validity. It reflects the practice in 4 different countries. Due to complete follow up of all patients in the study population with censoring at the time of death, emigration, or at the end of follow up, selection bias is unlikely. Additionally, only patients who received an Oxford III UKA with the diagnosis OA were selected (Fig. 1). We limited the analyses to the latest time period from 2000, excluding older implants and techniques. Using previously described methods of analysing the impact of procedure volume also strengthen the study [20, 22, 24, 25]. The advantage of analyzing each year separately is the reflection of the procedure volume that particular year.

Revision was less likely in older patients compared to the younger in our study. Other studies have shown that young patients experience an increased risk of revision after UKA compared to older patients [21, 26–28]. W-Dahl et al. [29] and Liddle et al. [21] also found that older patients had the greatest benefits and the lowest revision rates. In addition, UKA has been associated with lower rates of morbidity and mortality compared to TKA [30]. Sweden had the best implant survival of all the 4 countries. This could be a result of longer training of Swedish surgeons, starting unicompartmental knee arthroplasty surgery and a knee arthroplasty register before the other Nordic countries, and thereby gaining more experience. Sweden differs from the other nations with less than 50% of the implanted UKAs being Oxford and thus their learning curve could be improved by surgical experience performing other types of UKA. Denmark had inferior results compared to the other countries and contributed to the majority of patients in high volume hospitals (52% in the ≥44 group). We performed sensitivity analysis with and without data from Denmark. The tendency in the results for the volume groups did not change excluding Denmark. Denmark also has poorer results in the low volume groups. The cause of poorer results in Denmark is not possible to verify, but learning curve, threshold for revision and patient selection could be explanation factors. Theoretically, an increase in inexperienced surgeons implementing a new technique could initially lead to many revisions, but if continued, an expected improvement should occur. This could also explain the deteriorating results in the last time period.

Analyses of specific revision causes revealed a higher risk of revision for unexplained pain in low volume hospitals as compared to higher volume hospitals. We found minor differences for the other revision causes (Table 4). Baker et al. found that while more unicompartmental knee implants than total knee implants were revised for unexplained pain, when these revisions for unexplained pain were discounted, unicompartmental knee arthroplasty still had a significantly greater risk of revision from other reasons than did total knee arthroplasty [31]. However the numbers of revisions in each group were too small to allow making any conclusions regarding the differences between the volume groups.

There has been an on-going discussion regarding the threshold for revision due to unexplained pain [32]. Similarly, the incidence of radiolucent lines at the bone-implant interface [33] could be misinterpreted as loosening by unexperienced Oxford-users, and thereby leading to unnecessary revisions. Nevertheless, in cases with concurrent pain or symptomatology, it could be argued that revision is motivated. These could be explanations to the differences in revision rates, suggesting a lower revision-threshold in low-volume users. However, even the highest volume hospitals could not match the outcomes reported by developers [6, 7, 34] or the results after TKA regarding revision rates [24, 35]. A retrospective independent sample of failures reported to the registers could be one approach to evaluate the indication for revision surgery and identifying critical errors in the primary surgical technique and patient selection. Precise surgical indications for both primary and revision surgery are still debated [8, 22]. Furthermore, whether emphasis should be put on the higher revision rates of UKA compared to TKA or the lower risk of postoperative death and complications comparing UKA to TKA is also important to take into consideration [35].

Limitations to the study may be unmeasured factors such as decision-making regarding pre-operative radiographic changes leading to primary indication for surgery [36]. In addition, information on life style factors and physical activity was not available. The selection of patients considered suitable for UKA surgery is debatable regarding radiographic findings, age and BMI [8, 22]. Only hospital procedure volume was available for analysis in the NARA database, surgeon caseload and experience were not available. Theoretically, a high volume surgeon in a high volume center would gain the best results according to a systematic review regarding surgery volume [37]. However, the volume of a center had an equal if not greater effect on patient outcome than surgeon volume. Categorization of the volume exposure assumes that any (unspecified) hospital-level effects (e.g. the care that patients within a specific hospital receive, independent of volume) are trumped by a potential volume effect on revision rates. The analyses in this study are limited to the cemented medial Oxford III UKA and may limit the generalizability of the results to be valid for other UKA implant types.

## Conclusion

Hospitals performing ≤11 Oxford III UKA per year had a higher risk of revision, and were more likely to perform revisions due to unexplained pain.

## Abbreviations

CI: Confidence Interval
HR: Hazard Ratio
IQR: Interquartile range
K-M: Kaplan Meier
N: numbers
NARA: Nordic Arthroplasty Register Association
NJR: National Joint Registry of England and Wales
OA: Osteoarthritis
Ref: Reference
TKA: Total Knee Arthroplasty
UKA: Unicompartmental Knee Arthroplasty
